# Reallocating Time Spent in Physical Activity, Sedentary Behavior and Its Association with Fear of Falling: Isotemporal Substitution Model

**DOI:** 10.3390/ijerph19052938

**Published:** 2022-03-03

**Authors:** Pengfei Ren, Xianliang Zhang, Litao Du, Yang Pan, Si Chen, Qiang He

**Affiliations:** 1 School of Physical Education, Shandong University, Jinan 250061, China; renpf1997@126.com (P.R.); xlzhang@sdu.edu.cn (X.Z.); tao7221@outlook.com (L.D.); panyang@sdu.edu.cn (Y.P.); 2 School of Nursing and Rehabilitation, Cheeloo College of Medicine, Shandong University, Jinan 250012, China

**Keywords:** fear, fear of falling, physical activity, secondary behavior, isotemporal substitution

## Abstract

The aim of the current study was to provide new evidence for the associations between physical activity (PA), sedentary behavior (SB), and fear of falling (FOF) by investigating the impact of replacing 30 min SB with both light-intensity PA (LPA) and moderate-to-vigorous PA (MVPA) on FOF in older Chinese women. Cross-sectional data from a Physical Activity and Health in Older Women Study (PAHIOWS) were analyzed for 1114 Chinese community-dwelling older women. Variables of focus were demographics, FOF, objectively measured PA and SB. Three different logistic models were used to examine the associations between PA, SB, and FOF (a single parameter model, a partition model and an isotemporal substitution). The results showed that reallocating 30 min/day of MVPA by SB was significantly associated with higher FOF (OR = 1.37; 95%CI: 1.04–1.79; *p* = 0.024), reallocating 30 min/day of SB by MVPA was significantly associated with a reduction of FOF (OR = 0.73; 95%CI: 0.56–0.96; *p* = 0.024). No significant associations were found between FOF with reallocating other activities by LPA and vice versa (*p* > 0.05). Subgroup analysis showed the isotemporal-substituted effects of MVPA and SB on FOF were stronger in older women with fall experience. In conclusion, the current findings showed that the increase of MVPA engagement and reduction of SB engagement may be most beneficial for FOF management and should be involved in public health guidelines, especially for older women with fall experience.

## 1. Introduction

Fear of falling (FOF), which was defined as low perceived self-efficacy and confidence at avoiding falls during essential, nonhazardous activities of daily living [1], has been clearly regarded as one of the most important and potentially modifiable threats to autonomy in older adults [2]. FOF is a contributor to many adverse health outcome, such as decreasing physical function, more frequent falls, more severe frail and rising mortality, especially in older women [3,4,5,6]. A prospective study of older adults identified that a sedentary or physically inactive lifestyle was a potential and vital predisposing factor for FOF [7].

Physical activity (PA) and sedentary behavior (SB) are independent determinants and major modifiable factors of many chronic diseases [8,9]. Both the 2020 Global Recommendations on PA and SB for Health updated by the World Health Organization and the PA Guidelines for Americans profoundly emphasized that PA is encouraged and SB should be limited among all age group to beneficially impact on the population health [10,11]. However, limited studies have investigated the associations between PA, SB lifestyle, and FOF severity while the inconsistent conclusions were constrained by PA and SB measurement methods, adjustment of confounding factors, as well as sample characteristics [12,13,14,15]. In addition, none of these studies has focused on benefits of PA, SB intensity, and time reallocation on the results due to limitations of analysis methods.

Isotemporal substitution models have been a new statistical analysis to investigate the effects of time engaged in different activities and varying displacement of other activities on health factors in recent years [16]. Considering that a day is a constant 24 h, the increase in time spent in one type of activity requires the reduction in time in other types of activity. Isotemporal substitution models take account for the finiteness of the time and help understand that daily behaviors (PA and SB) are co-dependent rather than separable. This is crucial for the individualized management of FOF as well as other health-related factors in older adults and may offer new insights for future public health recommendations for PA and SB.

Therefore, the aim of the current study was to provide new evidence for the associations between PA, SB, and FOF in older women by investigating the impact of replacing 30 min SB, light-intensity PA (LPA) and moderate-to-vigorous PA (MVPA) by one another on FOF in older women using isotemporal substitution models. We hypothesized that the differential impact of replacing a specific activity by increasing another on FOF is depending on the nature of successor.

## 2. Materials and Methods

### 2.1. Participants

Data were derived from the baseline survey of the Physical Activity and Health in Older Women Study (PAHIOWS). The study followed a positivism philosophy which identify associations through quantitative approaches to verify priori hypotheses. This is a cross-sectional study which used a convenience sampling method to recruit participants from March to June 2020. A total of 1370 older women were volunteered for assessments, participants who meet the inclusion criteria below were involved in the final analysis: (1) community-dwelling women aged 60~70, (2) able to communicate freely, (3) without cognitive impairment, and (4) willing to sign an informed consent form. Of all the 1370 older women, 195 women had insufficient accelerometer wear time, 56 women did not complete the questionnaire assessment, and five women did not finish the balance ability test. Overall, 1114 participants were included in the data analysis. To investigate whether fall experience affects isotemporal-substituted effects, participants were also analyzed in sub-cohort with or without fall experience. This study was approved by the Ethics Committee of the School of Nursing and Rehabilitation, Shandong University, China (2021-R-001). All participants were informed of the purpose, methods, expected benefits, potential dangers of the study before measurement and that they have the right to quit the study at any time during measurement. Researcher have ensured psychological, physical, and privacy security of participants throughout the data collection and analysis phase.

### 2.2. Measures

#### 2.2.1. Fear of Falling

The study used Chinese version of the Activities-Specific Balance Confidence (ABC) to assess FOF, which is based on Bandura’s theory of self-efficacy [17]. Older women were instructed to report their level of self-confidence when performing specific activities (for example, standing on tiptoes and reaching for something above head, walking up or down a ramp, and walking outside on icy sidewalks) without becoming unsteady by choosing percentage points on the scale from 0% (have no confidence at all) to 100% (with plenty of confidence). If they have never did the specific activity ever yet, they were required to imagine how confident they would be if they had to do. ABC is a mature and structured scale compared with some previous studies that used a single-item question (“Are you afraid of falling?”). In addition, it is more suitable for older adults with slightly higher activities involved in daily living. Participants with a higher ABC score had a higher level of subjective confidence in their balance and were regarded as at a lower FOF level. In this study, the 75th percentile of ABC score, corresponding to 88.13, was used as the cutoff point for classification of FOF. Subjects below the 75th percentile of ABC scores were regarded as the low FOF group.

#### 2.2.2. Physical Activity and Sedentary Behavior

Actigraph wGT3X-BT (Actigraph, Pensacola, FL, USA) accelerometer was used to perform PA and SB assessment [18]. Participants were advised to wear the accelerometer on their hip for 7 consecutive days, at all times except for night sleeping, showering, or swimming, and to maintain their normal lifestyle during these days. The sampling rate of raw data was 30 times per second. Raw data were transformed into counts of movement of 60 s epoch length using Actilife software 6.13.4 before analysis. Non-wearing time was defined as at least 90 consecutive minutes of 0 counts, with an allowance of a maximum of 2 min of counts between 0 and 100 count per minute (CPM) assessed by accelerometer [19]. Participants’ data with a wear time ≥ 10 h per day and ≥4 days were considered valid for further analyses [20]. Cutoff points defined by Freedson were used to classify sedentary time (SB), light-intensity PA (LPA), and moderate-to-vigorous intensity PA (MVPA) as follows: SB (0 to 99 CPM); LPA (100 to 1951 CPM); and MVPA (≥1952 CPM) [21]. Total wearing time which equals to SB plus MVPA time plus LPA, was constructed by automatic algorithms of accelerometers and daily data were calculated.

#### 2.2.3. Covariate Variables

Data on sociodemographic variables were collected by self-reporting individual interviews, including age, education status (primary school or less, middle school, bachelor’s degree or higher), living alone (yes, no), current drinker (yes, no), fall experience (0 fall, ≥1 fall), and number of medications taking (<5, ≥5). The nutritional and cognitive status of the included participants were assessed using short-form mini-nutritional assessment (MNA-SF) [22] and mini-mental state examination (MMSE) [23], respectively. Body mass index (BMI, kg/m^2^) and muscle mass (kg) were measured by multi-frequency bioimpedance analysis using the Tanita MC-180 (Bailida Co., Tokyo, Japan) [24]. Balance capacity was assessed using the Super Balance III Static Balance Test System (Acmeway, Beijing, China) [25]. Subjects were instructed to complete several static moves and holding each position for 30 s on a force platform, the trajectory and velocity data of center of gravity movement were obtained and calculated for balance stable coefficient.

### 2.3. Statistical Analyses

Descriptive characteristics were presented as means and standard deviations (SDs) or as numbers and percentages (%). Differences between the low FOF group and the high FOF group were assessed using the Mann–Whitney test, Fisher’s exact test, chi-square test, and trend chi-square test depending on the type of variables. Three different logistic models were used to examine the associations between PA, SB, and FOF (a single parameter model, a partition model, and an isotemporal substitution). The single parameter model assessed the associations of each single activity component (SB, LPA, and MVPA) with FOF separately with the adjustment of total accelerometer wearing time. The partition model partitioned total time among its components (SB, LPA, and MVPA) and assessed the effect of increasing one activity type (rather than substituting an activity type) while holding other activity type constant without the adjustment of total accelerometer wearing time. The isotemporal substitution assessed the effects of replacing one activity time with another physical activity type for the same amount of time (e.g., to replace SB with the same amount of time of MVPA, by taking SB out of the model while holding total time constant) [16]. Before running these models, the accelerometer-measured continuous variables were rescaled so that a one-unit increase reflected 30 min/day for each variable. Odds ratios (ORs) and 95% confidence intervals (CIs) were estimated for 30-min/day increments in SB, LPA, and MVPA. To further determine whether the isotemporal-substituted effects is different due to fall experience, we performed isotemporal substitution models in both full cohort and sub-cohort (older women with or without fall experience). Analyses in full cohort were adjusted for age, education, living alone, current drinker, and number of medications taking, nutritional status, MMSE score, BMI, muscle mass, fall experience, and balance ability. What is worth mentioning is that we also examined the MVPA-fall experience and SB-fall experience interaction effects on FOF, respectively, it turned out that neither of these interactive effects were significant hence details of the result were not reported in this study. All analyses were conducted using Stata version 16 (StataCorp, College Station, TX, USA). The statistical significance was set at *p* < 0.05 in two-sided tests.

## 3. Results

A total of 1114 older women were enrolled in the final analysis with a mean age of 64.9 years old. Table 1 showed the characteristics of participants. An amount of 212 older women (19%) were identified as low FOF group. More than a quarter of older women (315) have a fall experience equal to or more than once. On average, these 1114 older women spent about 9.1 h, 5.1 h, and 0.5 h a day in engaging in SB, LPA, and MVPA, respectively. Significant differences were observed between low FOF group and high FOF group in age, education status, fall experience, nutritional status, BMI, balance ability, and MVPA time. No difference was observed in other variables between groups.

Table 2 showed the isotemporal-substituted effects of PA and SB on FOF examined by multivariable-adjusted binary logistic regression in full cohort and subgroup. If the row and column headers correspond to the same variable, the cell contents at the intersection of rows and columns were expressed as “dropped”. In the single-parameter model, each 30 min/day increase of SB was significantly associated with higher FOF (OR = 1.08; 95%CI: 1.01–1.15; *p* = 0.025), while each 30 min/day increase of MVPA was significantly associated with lower FOF (OR = 0.71; 95%CI: 0.54–0.93; *p* = 0.013), no associations was found between LPA and FOF (OR = 0.94; 95%CI: 0.88–1.01; *p* = 0.084). In the partition model, a significant association was observed between MVPA and FOF in older women (OR = 0.75; 95%CI: 0.57–0.98; *p* = 0.039) with the independence of SB, LPA, and other confounders. In the full cohort, isotemporal substitution showed that reallocating 30 min/day of MVPA by SB was significantly associated with higher FOF (OR = 1.37; 95%CI: 1.04–1.79; *p* = 0.024). When this volume of MVPA substituted the same volume of SB, we found a significant association with a reduction of FOF (OR = 0.73; 95%CI: 0.56–0.96; *p* = 0.024). No significant associations were found with FOF after using 30 min/day LPA substituting the same volume of MVPA or SB and vice versa (*p* > 0.05). Subgroup analysis showed significant associations with FOF after replacing 30 min/day MVPA by SB and vice versa in older women with fall experience (*p* < 0.05). No such findings were seen in those without fall experience.

## 4. Discussion

This study investigated the associations of time reallocating of SB, LPA, and MVPA with FOF among a cohort of community-dwelling older women. For this purpose, we applied isotemporal substitution analysis to understand the potential benefits in regard to FOF of reallocating time spent in one type of activity (SB, LPA, MVPA) to another. In our study, 30 min/day MVPA substituted for a same volume in SB was significant associated with lower odds of FOF in older women, while using 30 min/day SB to replacing the same volume of MVPA demonstrated a strongly relationship with the increment of FOF level. These potential effects were even stronger among non-fallers.

### 4.1. Associations of Each Single Activity Component with FOF

In our study, time of older women spent in SB per day was close to OPACH Study which observed a mean SB time of about 9.2 h in community-dwelling women also using Actigraph triaxial accelerometer; however, when it comes to MVPA, the value was smaller than OPACH study (0.9 h) [26]. Time of older women spent in LPA in the current study was a bit longer than in OPACH Study (4.7 h) [27]. The differences may be due to different lifestyle between older Chinese women and older Western women. In regard to results of associations between each single activity component with FOF, we found that with the absence of mutual adjustment of single activity components each other, both 30 min/day increase of SB and MVPA were significantly associated with FOF. These are in accordance with Canever et al. [28] and Whipple et al. [29], which found that participant with more ST and less MVPA tended to report greater FOF. When all activity components were included in the model, we found that only 30 min/day increase of MVPA was independently associated with FOF, with a 25% lower FOF odds. This is inconsistent with Rosenberg et al. which found that higher ST was still associated with FOF after adjusting MVPA. We suspect that this may be due to the characteristics of the participants in Rosenberg et al. as their average age was 84 years old which is way older and more inactive than participants in the current study. According to our study, MVPA may altered the effect of SB on FOF, which means the adequate participation of MVPA may benefit older women’s FOF management no matter how long they spent in ST.

### 4.2. Isotemporal-Substituted Effects of MVPA on FOF

Previous evidence has shown some public health implications due to isotemporal substitutions of PA and SB. Several studies have highlighted the beneficial effects of replacing inactive behavior by equal volume with PA for cardio-metabolic risk factors [30,31,32]. Other studies also revealed that the isotemporal-substituted effects of PA and SB were associated with chronic diseases [33,34]. A systematic scoping review of 56 isotemporal substitution studies showed that current evidence almost consistently suggested that reductions in mortality risk are greater when time spent in SB is replaced with higher intensities of PA, the results to other health-related factors are similar [35]. The review also held that the studies which using isotemporal substitution models to assess the effect to mental health outcomes are scarce. As mentioned before, engaging in MVPA showed a statistical and independent relationship with FOF by demonstrating an increment of 30 min/day are associated with 25% reduction of FOF odds. In the further isotemporal substitution model, we found that replacing 30 min SB per day by the same volume of MVPA was associated a 27% lower OR of FOF. Our study has investigated isotemporal-substituted effects of PA and SB on FOF for the first time and supports the accumulating evidence of a close association between MVPA and health-related factors by emphasizing a vital role of MVPA replacing SB on FOF reduction in full cohort. FOF is a psychological phenomenon that occurs as a result of anxiety and worry about falls occurring. Evidence have already revealed that higher intensity of PA was associated with the reduction of anxiety [36]. In addition, spending more MVPA time in daily life help improve muscle strength, balance, and coordinate ability, which further build a stronger body. Over time, older women could feel positive changes in their body and become more confident to keep balance when performing slightly more difficult activities in daily life.

### 4.3. Isotemporal-Substituted Effects of SB on FOF

With regarding to SB, most of the previous isotemporal substitution model studies have focused on the health effects of replacing SB with other activities. However, the negative effects of substituting SB for active activities should also be illustrated to alert the public. Several studies have demonstrated that the substituting of SB for other activities have been found to play a negative role in health factors, including quality of life [37], fatigue [38], physical function [39], metabolic risk [40], and mortality risk [41]. Our study observed that a 30 min increment of SB per day was significantly associated with 8% increment of OR in single-parameter model. However, when MVPA was added in the partition model, the negative role of SB on FOF was not statistical anymore. Therefore, the engaging of sufficient increment of MVPA time may counteract the negative effects of ST on FOF. Our result of isotemporal substitution model further deepens the analysis of the relationship of SB and FOF by demonstrating that replacing 30 min MVPA per day with the same volume of SB induced a higher 37% OR of FOF in full cohort. In fact, the relationship between SB and FOF is possibly bidirectional as FOF may also lead activity restriction of older people [42]. Based on our finding, we believe that focusing on avoiding unnecessary SB may be effective to break this vicious cycle.

### 4.4. Isotemporal-Substituted Effects of LPA on FOF

Although serval previous studies have showed the health benefits of substituting of LPA for other activities [34,43], the results of the effect on other health-factors remained controversial. Some studies suggested that the substituting of higher-intensity PA to other activities may induce a better effect than LPA [16,44]. In our study, no LPA-FOF association was found significant whether in the single-parameter, partition, or isotemporal substitution models. For older women, LPA accounts for most of the daily PA. In our study, older women spent approximately 5 h per day engaging in LPA, and no significant difference have found between high FOF group and low FOF group. With such a long-time LPA per day, it is possible that the increment may not effective enough to provide the improvement of health. Especially in older women with FOF, they are afraid to engaging in higher-intensity activities, so they limit activities to sedentary and low intensity. Our finding echoed the previous studies that the substituting of MVPA is more effective.

### 4.5. Subgroup Analysis

In our subgroup analysis, we found similar effect on FOF when replacing activities by one another in older women who experienced a fall as in full cohort, that is, the substituting of MVPA and SB for each other revealed significant associations with FOF by showing a 40% lower FOF odds after replacing SB with the same volume of MVPA and a 66% higher FOF odds after replacing MVPA with the same volume of SB in women who experienced a fall, which showed same trend but greater change of odds compared with the full cohort. While no significant effects on FOF were found after substituting PA and SB in older women without a fall experience. The distinct result of isotemporal substitution model due to population subgroup have also been demonstrated in other health-related factors. Song et al. found that beneficial associations with pain by substituting moderate PA for an equivalent time of other behaviors were particularly pronounced in individuals without restless sleep, but not in those with restless sleep [33]. Our similar finding further supports that the isotemporal-substituted effects of PA and SB diverse by individual characteristics. Specifically, the potential effects of replacing MVPA and SB with each other on FOF were stronger among older women with fall experience. Evidence has shown that experiencing a fall increases the likelihood of developing FOF [3]. In our study, the proportion of older women who experienced falls in the high FOF group was significantly higher than that in low FOF (40.1% versus 25.5%), which may have explanatory power because women with fall experience may have suffered severe FOF and thus may be more sensitive to the isotemporal-substituted effects of MVPA and SB. Overall, this finding reminds older women with fall experience that engaging in MVPA and avoiding unnecessary SB may provide a benefit to the reduction of FOF.

### 4.6. Strength and Limitations

This is the first study performing isotemporal substitution model to investigate the associations of objectively measured PA, SB, and FOF in older women. Several interventions have been shown to effectively reduce FOF especially exercise intervention, such as moderate-intensity home-based exercise and community group-based Tai Chi [45]. We affirm the role of targeted exercise programs on FOF, however we also believe that the promotion of spontaneous activity in a natural environment may be much easier for older women to involve in compared with planned exercise which requires instruction. However, as mentioned before, this is a cross-sectional study which is not able to prove a causal link, longitudinal study designs are needed in the future. In addition, we only assessed daytime activities when older women were awaking. Hence, we were not able to consider all 24 h daily movement-related behaviors including sleep in the isotemporal substitution model. In addition, this study used a convenience sampling method which may not be able to fully represent a well-defined population.

## 5. Conclusions

Our findings, stemming from a study with an objectively measurement PA and SB, support the hypothesis of the positive associations between more PA involvement and FOF management, in the form of increments in MVPA and SB reduction in older women, especially among those who experience falls. LPA may have marginal effects on FOF but is not statistical. The current findings add to evidence that MVPA and SB may have isotemporal-substituted effects on mental health outcomes. Larger long-term prospective study and randomized controlled trials are needed to further illustrate the isotemporal-substituted effect of PA and SB on more health-related factors in order to provide more targeted guidance for public health.

## Figures and Tables

**Table 1:** Characteristics of participants by FOF classification (*n* = 1114; collected from March to June 2020 in Yantai, China).

Variables	Total	FOF Group Score		*p*
		High (*n* = 212)	Low (*n* = 902)	
Age, years	64.9 ± 2.8	65.4 ± 2.8	64.8 ± 2.8	0.005
Living alone, *n* (%)	126 (11.3)	18 (8.5)	108 (12.0)	0.184
Current drinker, *n* (%)	105 (9.4)	21 (9.9)	84 (9.3)	0.794
Education, *n* (%)				
Primary school or lower	132 (11.9)	37 (17.5)	95 (10.5)	0.004
Middle school	779 (69.9)	145 (68.3)	634 (70.3)	
Bachelor degree or higher	203 (18.2)	30 (14.2)	173 (19.2)	
No. of fall experience ≥ 1, *n* (%)	315 (28.2)	85 (40.1)	230 (25.5)	<0.001
No. of medications taking ≥ 5, *n* (%)	14 (1.3)	5 (2.4)	9 (1.0)	0.160
MNA-SF Score < 11, *n* (%)	32 (2.9)	12 (5.7)	20 (2.2)	0.011
Muscle mass, kg	38.7 ± 2.9	38.7 ± 2.8	38.7 ± 3.0	0.982
BMI				
Underweight (<18.5)	12 (1.0)	1 (0.5)	11 (1.2)	<0.001
Normal (18.5 ≤ BMI < 24)	376 (33.8)	50 (23.6)	326 (36.2)	
Overweight (≥24)	726 (65.2)	161 (75.9)	565 (62.6)	
MMSE score	26.7 ± 1.4	26.5 ± 1.5	26.7 ± 1.4	0.056
ABC score	92.7 ± 9.2	77.2 ± 10.2	96.4 ± 3.2	<0.001
Balance stable coefficient	12.5 ± 1.9	12.2 ± 2.1	12.6 ± 1.9	0.009
SB, (min/day)	548.7 ± 118.1	564.9 ± 128.6	544.9 ± 115.3	0.056
LPA, (min/day)	306.5 ± 71.2	300.1 ± 68.9	308.0 ± 71.7	0.396
MVPA, (min/day)	32.7 ± 19.3	28.5 ± 20.4	33.7 ± 18.9	<0.001
Wear time, (min/day)	888.0 ± 118.6	893.5 ± 130.7	886.7 ± 115.6	0.975

Mean ± standard deviation unless noted. ABC: activities-specific balance confidence scale; BMI: body mass index; FOF: fear of falling; LPA: light-intensity physical activity; MMSE: mini-mental state examination; MNA-SF: short-form mini-nutritional assessment; MVPA: moderate-intensity physical activity; *n*: number; SB: sedentary behavior. *p*: Probability values for differences between high FOF and low FOF group.

**Table 2:** Isotemporal substitution models of PA and SB on FOF (*n* = 1114; collected from March to June 2020 in Yantai, China).

Models	SB		LPA		MVPA	
	OR	95%CI	OR	95%CI	OR	95%CI
Single-parameter model						
	1.08	1.01–1.15	0.94	0.88–1.01	0.71	0.54–0.93
Partition model						
	1.02	0.98–1.06	0.97	0.91–1.04	0.75	0.57–0.98
Substitution model						
In full cohort, replacing 30-min/day of						
SB by	Dropped		0.95	0.89–1.02	0.73	0.56–0.96
LPA by	1.05	0.98–1.13	Dropped		0.77	0.57–1.02
MVPA by	1.37	1.04–1.79	1.30	0.98–1.74	Dropped	
In older women with fall experience, replacing 30-min/day of						
SB by	Dropped		0.91	0.81–1.03	0.60	0.37–0.97
LPA by	1.10	0.97–1.24	Dropped		0.66	0.39–1.11
MVPA by	1.66	1.03–2.69	1.52	0.90–2.54	Dropped	
In older women without fall experience, replacing 30-min/day of						
SB by	Dropped		0.97	0.88–1.06	0.84	0.60–1.17
LPA by	1.03	0.95–1.13	Dropped		0.87	0.61–1.24
MVPA by	1.19	0.86–1.66	1.15	0.81–1.65	Dropped	

CI: confidence interval; FOF: fear of falling; LPA: light-intensity physical activity; MVPA: moderate-intensity physical activity; OR: odds ratio; SB: sedentary behavior.

## Data Availability

Not applicable.
